# The unbuilt environment: culture moderates the built environment for physical activity

**DOI:** 10.1186/s12889-016-3866-3

**Published:** 2016-12-05

**Authors:** Andrew J. Perrin, Neal Caren, Asheley C. Skinner, Adebowale Odulana, Eliana M. Perrin

**Affiliations:** 1 Department of Sociology, College of Arts and Sciences, University of North Carolina, CB#3210, 155 Hamilton Hall, Chapel Hill, NC 27599-3210 USA; 2 The Duke Clinical Research Institute, 2400 Pratt Street, Office 8047, Durham, NC 27705 USA; 3 Medical University of South Carolina, 165 Ashley Avenue, MSC 561, Charleston, SC 29425 USA; 4 Department of Pediatrics, School of Medicine, University of North Carolina, 231 MacNider, CB#7225, Chapel Hill, NC 27599-7225 USA

**Keywords:** Obesity, Culture, Built environment, Physical activity, Rural

## Abstract

**Background:**

While research has demonstrated a link between the built environment and obesity, much variation remains unexplained. Physical features are necessary, but not sufficient, for physical activity: residents must choose to use these features in health-promoting ways. This article reveals a role for local culture in tempering the effect of the physical environment on physical activity behaviors.

**Methods:**

We developed Systematic Cultural Observation (SCO) to observe place-based, health-related culture in Lenoir County, NC (population ~60,000). Photographs (*N* = 6450) were taken systematically from 150 most-used road segments and geocoded. Coders assessed physical activity (PA) opportunities (e.g., public or private activity spaces, pedestrian-friendly features) and presence of people in each photograph.

**Results:**

28.7% of photographs contained some PA feature. Most were private or pedestrian; 3.1% contained public PA space. Only 1.5% of photographs with any PA features (2% of those with public PA space, 0.7% of those with private) depicted people despite appropriate weather and daylight conditions.

**Conclusions:**

Even when PA opportunities existed in this rural county, they were rarely used. This may be the result of culture (“unbuilt environment”) that disfavors physical activity even in the presence of features that allow it. Policies promoting built environments designed for healthy lifestyles should consider local culture (shared styles, skills, habits, and beliefs) to maximize positive outcomes.

## Background

Research on the built environment has demonstrated significant effects of neighborhood physical characteristics on health-related practices and outcomes, particularly with respect to obesity. Physical features constitute one important mechanism by which locality affects such practices and outcomes; however, focusing only on physical features may lead to missing other mechanisms. Using a novel method for measuring health-related, place based culture, we demonstrate that culture—the styles, skills, habits and beliefs of a community—affects the ways the built environment influences health-related practices.

### Obesity: patterns and causes

Overweight and obesity are very common conditions and increasing in prevalence. At least 64.8% (68.5% by another account [1]) of US adults are overweight (35.4%) or obese (29.4%) [2], with prevalence as high as 82% in African-American women, the most vulnerable group [1]. Childhood and adolescent overweight is also a major health problem with a large burden of suffering. Based on the definition of childhood overweight recommended by the Centers for Disease Control and expert committees, the current prevalence of being overweight or obese is 32.2% among children 2–17 [3]. Becoming overweight can be rapid in young adulthood, especially for young African American and Hispanic women [4]. Both child and adult overweight and obesity are associated with major psychosocial and health consequences, including stigmatization, depression, metabolic syndrome, hyperlipidemia, hypertension, and orthopedic problems.

The rapidly changing epidemiology of obesity suggests that the primary causes of changes in rates of obesity and being overweight are likely environmental. Thus efforts to prevent overweight and obesity should focus on modifying the environment, including both physical surroundings and culture [5]. Many factors of our “obesogenic” culture have been implicated, particularly in children and the transmission of behaviors and obesity from parents to children. Elements of culture may be transmitted differently in different races and ethnicities and in different local areas. Racial and ethnic differences in risk factors for obesity exist prenatally and in early childhood [6, 7], as do differences between local areas, counties, states, and regions. These likely signal effects of cultural exposures; for example, obesogenic and fat-stigmatizing messages were found to be very common in top-grossing children’s movies [8], one among many sources of cultural cues with health implications.

Another important body of research has demonstrated effects of the physical local environment, often termed the “built environment.” Many elements of local physical environments may affect individuals’ health-related decision making, both through constraining the options available and through guiding individuals toward choosing one option over another. The built environment can be thought of as physical characteristics of a community that affect activity or dietary behaviors [8]. For example, busy streets are a component of the built environment that may discourage walking, while sidewalks along those streets may encourage it [9]. Grocery stores and farmers’ markets may encourage healthier dietary choices, while high prices and food deserts may discourage them. Key to the concept of the built environment is the insight that opportunities for healthy choices are a necessary precondition for making those choices. Socioeconomic status (SES) disparities in access to physical activity (PA) facilities are an important factor in explaining SES disparities in overweight and obesity [10]. Environmental factors in the built environment that have been associated with higher rates of overweight and obesity include increased distances to recreational facilities, aesthetically unpleasant communities for physical activity, feeling unsafe with regard to crime and/or traffic, and the lack of attractive nonresidential destinations [11].

Research on the built environment is often focused on urban areas (i.e. in New York City [12]). However, rural areas experience higher rates of obesity and overweight than urban areas, and the reasons for this disparity remain an area of active research [13]. Elements of the physical environment that affect activity or dietary behaviors in rural areas may not be literally “built” -- open spaces for play, for example, can encourage physical activity, while country roads without built shoulders may discourage it [11]. Neighborhood parks and playgrounds have been shown to have a significant effect in reducing BMI and the risk of obesity among children [14]. Conversely, the prevalence of fast food restaurants is an independent risk factor for state and community obesity rates [15]. Overall, rural areas often present physical barriers to physical activity and other health behaviors [16].

### Conceptualizing and measuring local culture

While much research has demonstrated a link between the built environment and obesity rates, much community-level variation in obesity and overweight remains unexplained. The presence of physical features is a necessary, but not sufficient, condition for physical activity: residents must choose to use these features in health-promoting ways. A park, for example, may be used for sports; for group barbecues and picnics; or not at all. Each of these possibilities has distinct health implications, and the choices among them likely reflect, in part, local culture: the shared assumptions about the proper use (or nonuse) of the physical facility. Some research has assessed the “social environment” as an additional explanatory concept, but this has generally been conceptualized only as Socioeconomic Status ﻿(SES)SES and social network composition [17] or perceptions of social undesirability [18], stopping short of a sociologically robust conceptualization of culture.

Current research in sociology conceptualizes culture as a set of shared meanings among a defined group or community. These shared meanings both shape and are shaped by institutions—such as media, everyday talk, advertising, and the taken-for-granted expectations for daily behavior—that carry these meanings. Cultures work by providing their members with structures for interpreting and participating in social life by defining rules, strategies, and resources available in social settings. Contemporary frameworks understand culture as providing a repertoire of resources and guidelines, at once enabling and constraining the available choices for social action at particular conjunctures [19]. Individuals are commonly subject to several cultures that may be hierarchically nested or cross-cutting. For example, a highly-educated African American woman living in Kinston, North Carolina, has access to—and is constrained by—cultural repertoires related to American culture; regional cultures specific to the American Southeast; race-related culture by virtue of her African American heritage; and a variety of microcultures stemming from involvement in civic, religious, workplace, and leisure activities [20, 21].

As a shared repertoire of styles, skills, habits, and beliefs, *culture in the mind* promotes some kinds of action while inhibiting others. For example, a repertoire might favor sedentary activities and label outdoor exercise as elitist, urban, or otherwise distant. Such a repertoire tends to make it more unlikely that individuals holding it will engage in outdoor exercise and, potentially, other healthy uses of physical features. However, individuals also learn cultural cues from *culture in the world*: the set of messages in media, conversation, and other artifacts that they encounter in daily life. And these messages are formed in part through the repertoire in cultures of the mind. So cultural effects on health-related behaviors are the result of—and should be measured through—the cyclical interactions between culture in the world and culture in the mind.

Culturally-influenced causes such as the built environment [10, 17], socioeconomic status [22, 23], and social capital [24] all have important effects on health in general, and on obesity in specific. Similarly, individual attitudes toward diet and exercise have important effects [25–27]. However, neither individual attitudes nor structural features of communities is sufficient to explain obesity outcomes. Place-based culture— the shared beliefs, styles, skills, and habits of residents of particular areas —is a likely candidate both for moderating and mediating the effects of structural social realities on obesity and other health outcomes. It constitutes an “unbuilt environment” that, in combination with the built environment, may constrain and enable health-related behaviors. Research in cultural sociology suggests that individuals make decisions in settings structured by both physical (“built”) and cultural (“unbuilt”) factors [19]. Building on contemporary research in the sociology of culture, we examine the role of the “unbuilt environment” in moderating the relationship between the built environment and obesity. We sought to systematically and robustly assess-- in a county with a high prevalence of obesity--the physical features relevant to food and physical activity (the built environment) as well as the observed usage of these components by individuals (the unbuilt environment). In this study, we use a systematic cultural observation of a theoretically-relevant rural community and report on the presence of these environmental features [19].

## Methods

### Location

The study was conducted in Lenoir County, North Carolina, a rural county chosen because its obesity rate (34%; [28]) is among the highest in North Carolina (ranked 14^th^ most obese out of 100 North Carolina counties) and higher than predicted based on demographic characteristics of the population, based on the authors’ unpublished analysis of BRFSS data [28]. The county has a total population of approximately 59,000, of whom approximately 22,000 live in Kinston, the county seat. The entire county represents 400 mile^2^, and 23.7% of the population lives below the federal poverty level. The population is about 41% African American and 53% white, with the remainder other minorities including the fastest-growing group, Latino immigrants. Community data from 2007 indicate 52.6% of the population was female, 47.4% male. Per capita income in 2009 was $18,877, well below the state average of $24,547, and 20.1% of residents were below the federal poverty level compared to 16.2% for the state [29]. The county’s Gini coefficient (a measure of income inequality) was 0.4651, compared to a state average of 0.4463 and a national average of 0.4350 [30].

### Procedures

**Fig. 1:**
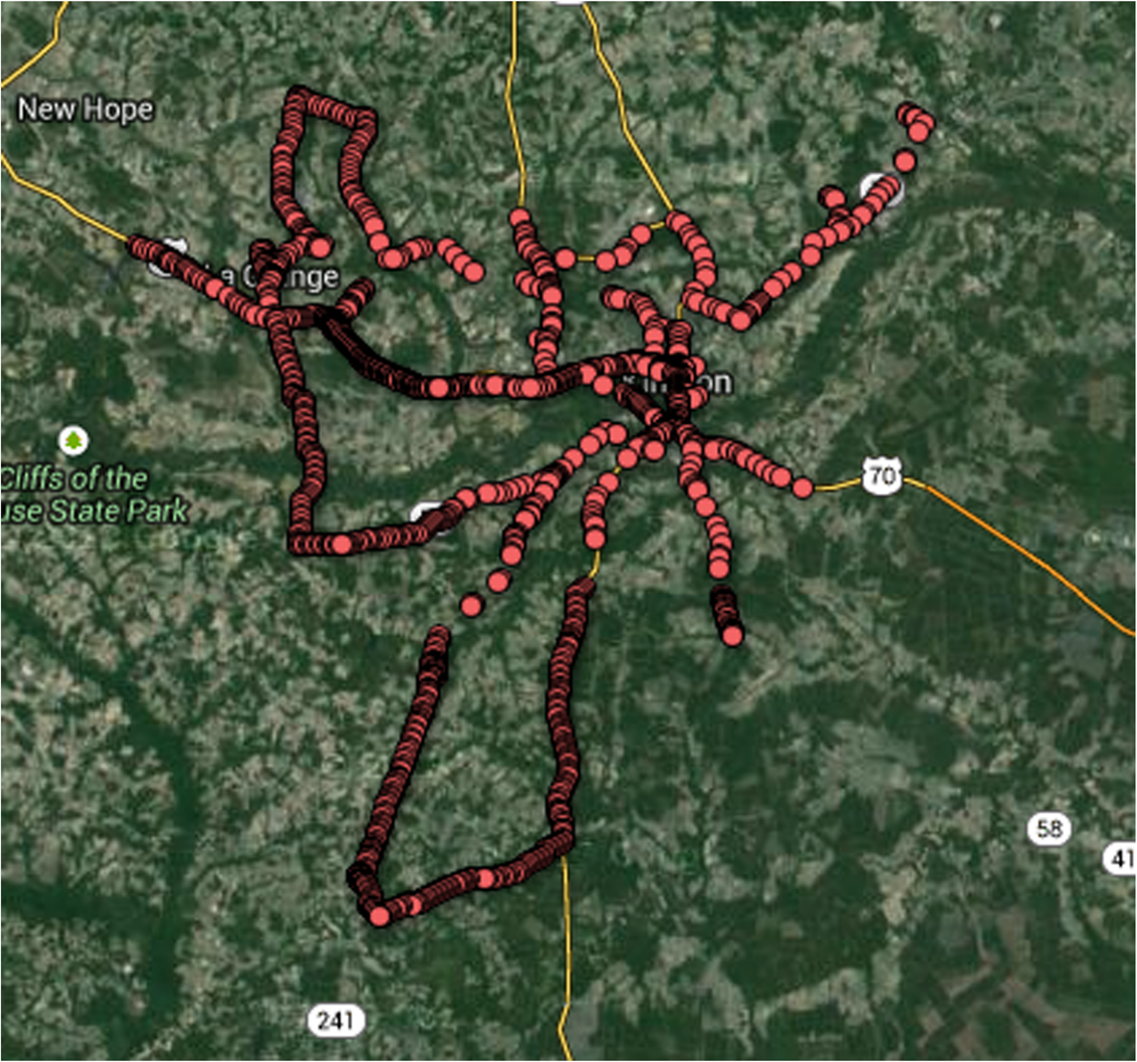
Map of all photographs taken and coded

We used Systematic Cultural Observation (SCO), a procedure we designed by adapting Systematic Social Observation (SSO [31]). In order to draw a sample of the most common roadways, we sampled 1000 pairs of census block groups, and computed driving directions between the points using the MapQuest Open Directions Application Programming Interface. From the directions, we computed the 150 most frequently used road segments in the county. We then computed driving directions that would enable us visit all of these points. The directions totaled 231.3 miles of roadway. In each of three cars, we mounted two cameras on the dashboard (one pointing left, the other right). The cameras were connected to a portable computer running the Linux operating system and a Global Positioning System (GPS) receiver. The computer collected images from each camera every three (3) seconds and tagged them with GPS coordinates. We removed duplicate images based on GPS coordinates. Such images occurred when the car was stopped for more than three seconds, such as at a traffic light or stop sign. Figure 1 shows the map of all locations where photographs were taken. Weather on the day of the photo capture (a Saturday) was sunny and 64° Fahrenheit, with no precipitation—an ideal day for outdoor physical activity. Review of local newspapers did not reveal any major competing events, and there was no significant sports event on television at the time.

### Measures and coding

After obtaining all photographs, our team coded each for the presence of a variety of characteristics. Using a coding tool we designed, seven different coders coded subsets of the photographs. For identifying physical features that foster physical activity, we began by developing a *de novo* codebook of anticipated types of spaces based on existing audit tools [31, 32] and our own observations. We then coded a small sample of images and, as a group, refined the coding scheme by discussing photographs and codes. We coded each photo for the presence and type of physical opportunity space. We defined physical activity space as any space that could theoretically be used for physical activity. For example, a grassy area beside a major road would not be considered physical activity space, but an open area around houses would be. We further defined physical activity space as either public or private. For example, the yard of a home would be considered private space, while a park would be considered public space. Physical activity opportunities were green spaces (public or private), sidewalks, play structures, buildings with play spaces other than houses (e.g., schools), and crosswalks. We also coded for the presence of people (not in motorized vehicles such as cars or trucks) in each photograph. We deliberately chose this very low standard—the sheer presence of a person—so as not to miss any possible physical activity in the photos. Even when a person is present, she may not be engaging in physical activity, but our generous standard establishes that she could be Table 1.

**Table 1:** Physical environment aspects facilitating physical activity and proportion with people

Type of Space	Percent of all Photos^a^ (*n* = 6450)		Percent of space that also included people	
	*N*	%	*N*	%
Any Physical Activity	1851	28.7	27	1.5
Public Activity Space	197	3.1	4	2.0
Private Activity Space	1105	17.1	8	0.7
Sidewalk	706	11.0	17	2.4
Park	23	0.4	0	0.0
Religious/Church	86	1.3	1	1.2
School	25	0.4	0	0.0
Crosswalk	112	1.7	4	3.6
Play Structure	28	0.4	0	0.0

^a^Column does not sum to 100% because some activity spaces meet criteria for two or more categories

Approximately 15% of photographs were randomly selected for coding by two or more coders to assess intercoder reliability (10 were coded by 4 or more coders; 86 by 3 coders; 811 by 2 coders). Agreement among raters was high, ranging from 84% for identification of space available for any physical activity, to 99-100% for presence of people, activities in which they were engaged, and the types of structures available. Mean Cohen’s kappa score for the 36 coded items was .66 which suggests substantial agreement among the coders. Kappa scores tend to be low, even with high agreement levels, for rare outcomes such as the ones we were identifying here. Hence Kappa scores are lower than one might expect given the high rate of agreement [33]. Coding discrepancies were resolved by both coders reviewing the photo together and agreeing upon the appropriate code.

### Analysis

We present analysis in two forms. First, we summarize the prevalence of types of physical activity opportunities and individuals observed in those spaces. Second, we present county maps with the distribution of these opportunities throughout the county and the presence of individuals at those locations.

## Results

A total of 6450 images were obtained, coded, and mapped. Almost 29% of all photos included some physical feature that would tend to encourage physical activity (Table 1). Most open spaces were private spaces, such as yards. Sidewalks were the most common type of built space. Still, 3.1% of photographs contain public physical activity spaces. The most striking finding in these data is that, across all types of physical activity spaces, very few people were using those spaces even given the near-perfect weather and opportunity.

**Fig. 2:**
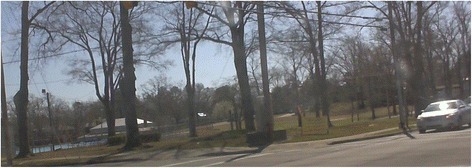
Emma Webb Park, Kinston, North Carolina, USA: physical activity opportunity with no users

Figure 2 contains a typical photograph showing physical activity space but no people using it. This particular photograph was taken at 11:27 am on a warm and sunny Saturday. This photograph depicts Emma Webb Park in Kinston, which includes a picnic shelter, softball field, and playground, along with open space, the city’s largest public swimming pool, and an active gymnastics program inside the building. Like many similar photographs, the streets are tree-lined and contain crosswalks and sidewalks—both features that facilitate pedestrian access and, therefore, outdoor exercise. Similarly, the park itself contains many facilities that could encourage physical activity. Nonetheless, this photo (like nearly all the others in the dataset) contains no people in the park itself or on the sidewalks or crosswalks.

Features that facilitate walking (sidewalks and crosswalks) had the most users, but only 2.4 and 3.6%, respectively, of these features had people using them. The rates are below 1% for most of the other features, and just 1.5% for all physical activity spaces.

**Fig. 3:**
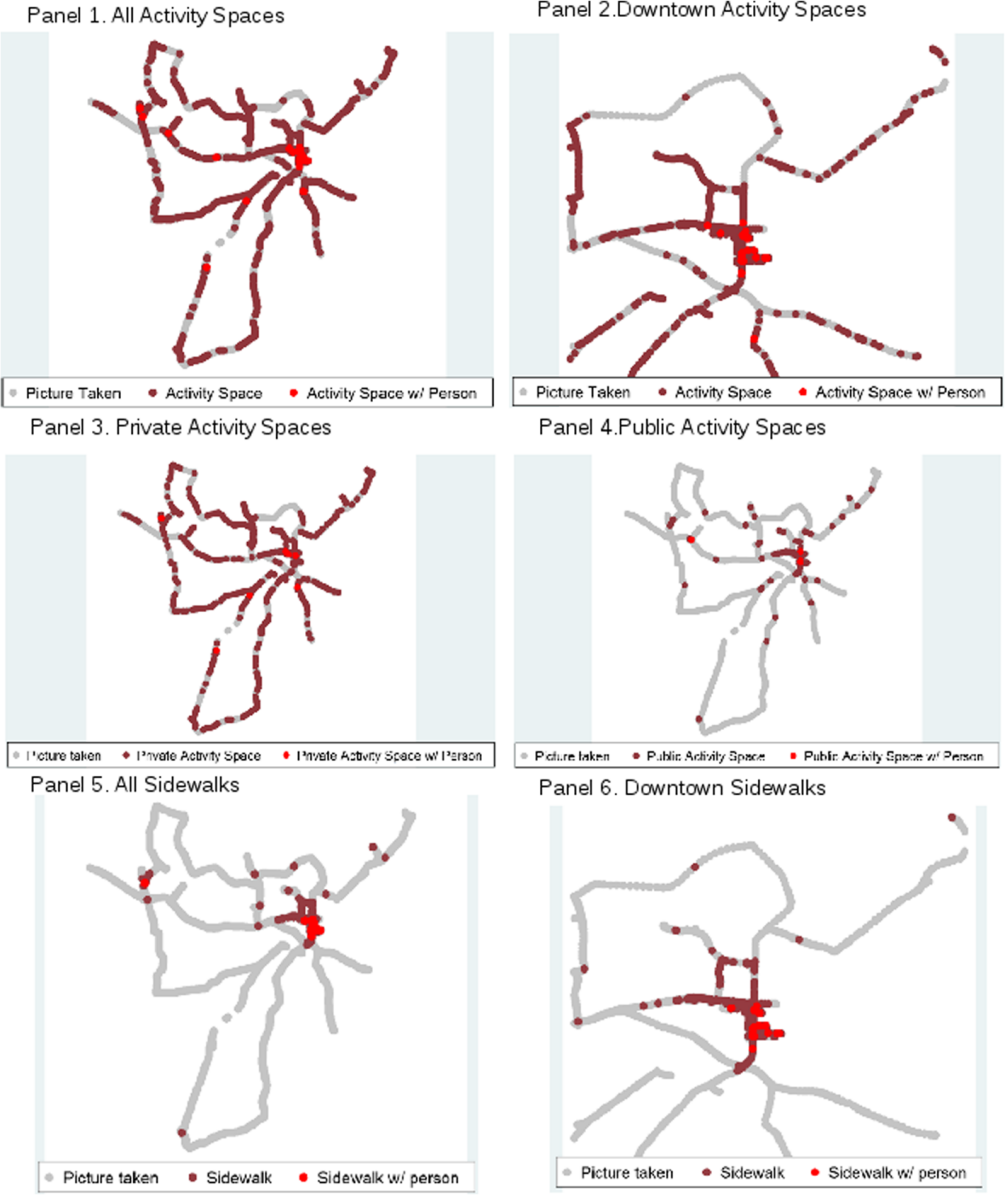
Physical activity opportunity spaces observed

Physical activity space was well-distributed throughout the county (Fig. 3, Panel 1). However, most of the few points with people present occurred in the downtown Kinston area (Fig. 3, Panel 2). The spread of public vs. private space differed throughout the county. Private physical activity space was seen throughout the county, primarily due to private residences (Fig. 3, Panel 3). Since physical activity spaces were ascertained via photo coding, we were able to determine whether there were people in private spaces as well as public. Public physical activity space was most heavily located in the downtown area (Fig. 3, Panel 4). Virtually all sidewalk spaces were in the downtown area (Fig. 3, Panel 5), though most of the sidewalks did not have people making use of them (Fig. 3, Panel 6).

## Discussion

Opportunities for physical activity are widely available throughout this poor, rural county: more than in other studies of similar rural southern places [16, 18]. Though most (1105) are private spaces, we identified 197 photos showing public activity spaces (just under one every two square miles). Even when activity opportunities exist, they are rarely being used, even on a weekend day with ideal weather. Public activity spaces, while still very underused, were nearly 3 times as likely to have people as were private spaces. We theorize that this underuse is the result of cultural patterns – an “unbuilt environment” -- that disfavor physical activity even in the presence of features that allow for it. In the specific case of Kinston and Lenoir County, culturally-sanctioned behaviors do not appear to include outdoor activities; the systematic photographs we took revealed that nearly all people were either driving or visiting commercial establishments through their parking lots. Further research, particularly focusing on how residents interpret and use physical features, can help understand the cultural barriers to the built environment’s effect on health.

Associations between aspects of the built environment and health outcomes may arise for several reasons. The assumption in much of the built-environment literature is what Hillier calls a “space syntax” paradigm, in which elements of the built environment encourage behaviors through enhancing some opportunities and foreclosing others [34]. The presence of a park, for example, and the lack of a fast-food restaurant make it more possible for a person to exercise outdoors and less possible for her to eat unhealthy food. Alternative mechanisms for these associations include selection effects, whereby individuals who prefer fewer parks and more fast food restaurants select neighborhoods with these features; and cultural effects, in which neighborhood features not only afford opportunities but also convey shared ideas about culturally-valorized eating and exercise patterns.

Culture may be an important, independent factor explaining health-related behaviors [21]. Previous research has indicated that availability of physical activity opportunities may be an important factor in obesity incidence and prevalence. Much of this research has focused on the availability of specific types of activity opportunities in the built environment, such as sidewalks and parks. However, the unbuilt environment is also an important component to availability of physical activity opportunities. To the extent that local places such as cities, counties, and states harbor health-related cultures, these cultures may help explain geographic variations in health outcomes. Further research using SCO and similar methods to measure culture can help shed light on these effects.

Current cultural sociology demonstrates that culture is best understood as a system of ideas, meanings, and mental representations (so-called “cultural repertoires” [35]) that simultaneously enable, guide, and constrain the strategic actions of individuals. Cultures work by providing their members with structures for interpreting and participating in social life by defining rules, strategies, and resources available in social settings. Contemporary frameworks understand culture as providing a repertoire of resources and guidelines, at once enabling and constraining the available choices for social action. Individuals engage in “conjunctural action” [19], deploying elements from their cultural repertoires to interpret and respond to situations that emerge in their environments.

Cultural sociology suggests that we look for the rules, strategies, and resources mobilized in groups and use specific contexts for cultural analysis. Various sociologists have shown that cultures linked particularly to parenting and social class predispose parents to particular behaviors that may affect their children’s health and achievement. Individuals are commonly subject to several cultures that may be hierarchically nested or cross-cutting [20, 21]. Neither individual attitudes nor structural features of communities are sufficient to explain health-related behaviors. Place-based culture—the ideas, meanings, and mental representations prevalent in an area—is a likely candidate both for moderating and mediating the effects of structural social realities on obesity and other health outcomes. There are other sources of culture as well—shared culture in larger units such as states, regions, and countries, as well as subcultures such as race and ethnicity. Local culture is particularly suited to geographically-observed differences such as the usage of the built environment.

### Limitations

This study presents the results of one day’s systematic observation, targeted for optimal conditions and establishing a “low bar” for physical activity. We cannot assess whether the day we observed was atypical for a reason unbeknownst to us. We infer the effect of local culture from the mismatch between the built environment and its use. A complete assessment of local culture would require further qualitative and quantitative work to understand the styles, skills, habits, and beliefs that promote some behaviors while reducing others. While we theorize that culture moderates the effect of the built environment on physical activity behaviors, we cannot formally test moderation using our research design; instead, we infer moderation from the lack of other explanatory processes.

## Conclusions

Our findings suggest that public health efforts to increase activity through the built environment will need to consider not just the physical environment but the unbuilt environment as well: the collection of shared styles, skills, habits, and values that make up local culture and affect the relationship between physical resources and actual behavior. Physical resources must be interpreted by actors as opportunities for healthier behavior in order for individuals’ conjunctural actions to benefit from such resources. Similarly, physical impediments may be amenable to reinterpretation more favorable to healthier behaviors. In such cases, increasing use of already-available physical activity opportunities through cultural change has the potential to serve as cost-effective means of leveraging the built environment. In both cases, the physical (“built”) environment needs to be considered in conjunction with its cultural (“unbuilt”) environments to understand the range of environmental effects on health-related behaviors. Future research can build on these findings to assess the specific cultural repertoires that constrain and enable the use of physical environment in health-promoting ways.

## Abbreviations

BRFSS: Behavioral risk factor surveillance system
GPS: Global Positioning System
PA: Physical activity
SCO: Systematic Cultural Observation
SES: Socioeconomic status
SSO: Systematic Social Observation

## References

[1] Ogden CL, Carroll MD, Kit BK, Flegal KM (2014). PRevalence of childhood and adult obesity in the united states, 2011-2012. JAMA.

[2] BRFSS Prevalence & Trends Data [http://www.cdc.gov/brfss/brfssprevalence]. Accessed 28 Nov 2016.

[3] Skinner A, Skelton JA (2014). Prevalence and trends in obesity and severe obesity among children in the united states, 1999-2012. JAMA Pediatr.

[4] McTigue KM, Garrett JM, Popkin BM (2002). The natural history of the development of obesity in a cohort of young US adults between 1981 and 1998. Ann Intern Med.

[5] Hill JO, Wyatt HR, Melanson EL (2000). Genetic and environmental contributions to obesity. Med Clin North America.

[6] Perrin EM, Rothman RL, Sanders LM, Skinner AC, Eden SK, Shintani A, Throop EM, Yin HS (2014). Racial and Ethnic Differences Associated With Feeding- and Activity-Related Behaviors in Infants. Pediatrics.

[7] Taveras EM, Gillman MW, Kleinman K, Rich-Edwards JW, Rifas-Shiman SL (2010). Racial/ethnic differences in early-life risk factors for childhood obesity. Pediatrics.

[8] Feng J, Glass TA, Curriero FC, Stewart WF, Schwartz BS (2010). The built environment and obesity: a systematic review of the epidemiologic evidence. Health Place.

[9] Davison KK, Lawson CT (2006). Do attributes in the physical environment influence children’s physical activity? A review of the literature. Int J Behav Nutr Phys Act.

[10] Gordon-Larsen P, Nelson MC, Page P, Popkin BM (2006). Inequality in the built environment underlies key health disparities in physical activity and obesity. Pediatrics.

[11] Boehmer TK, Lovegreen SL, Haire-Joshu D, Brownson RC (2006). What constitutes an obesogenic environment in rural communities?. Am J Health Promot.

[12] Rundle A, Diez Roux AV, Freeman LM, Miller D, Neckerman KM, Weiss CC (2007). The urban built environment and obesity in New York City: a multilevel analysis. Am J Health Promot.

[13] Penney T, Rainham D, Dummer T, Kirk S (2014). A spatial analysis of community level overweight and obesity. J Hum Nutr Diet.

[14] Fan M, Jin Y. Do neighborhood parks and playgrounds reduce childhood obesity? Am J of Agric Econ. 2013;96(1):aat047.

[15] Maddock J (2004). The relationship between obesity and the prevalence of fast food restaurants: state-level analysis. Am J Health Promot.

[16] Robinson JC, Carson TL, Johnson ER, Hardy CM, Shikany JM, Green E, Willis LM, Marron JV, Li Y, Lee CH (2014). Assessing environmental support for better health: active living opportunity audits in rural communities in the southern united states. Prev Med.

[17] Nelson MC, Gordon-Larsen P, Song Y, Popkin BM (2006). Built and social environments: associations with adolescent overweight and activity. Am J Prev Med.

[18] Li C, Chi G, Jackson R (2015). Perceptions and barriers to walking in the rural south of the United States: the influence of neighborhood built environment on pedestrian behaviors. Urban Des Int.

[19] Johnson-Hanks JA, Bachrach CA, Morgan SP, Kohler H-P, Hoelter L, King R, Smock P. Understanding family change and variation: Toward a theory of conjunctural action. Volume 5: Springer Science & Business Media; 2011.

[20] Perrin AJ (2006). Citizen Speak: The Democratic Imagination in American Life.

[21] Eliasoph N, Lichterman P (2003). Culture in interaction. Am J Sociol.

[22] Scharoun-Lee M, Kaufman JS, Popkin BM, Gordon-Larsen P (2009). Obesity, race/ethnicity and life course socioeconomic status across the transition from adolescence to adulthood. J Epidemiol Community Health.

[23] Hertzman C, Siddiqi A (2009). Population health and the dynamics of collective development.

[24] Hall P, Taylor RC (2009). Health, social relations and public policy.

[25] Davis AM, James RL, Curtis MR, Felts SM, Daley CM (2008). Pediatric obesity attitudes, services, and information among rural parents: a qualitative study. Obesity.

[26] Webb TL, Sheeran P (2006). Does changing behavioral intentions engender behavior change? A meta-analysis of the experimental evidence. Psychol Bull.

[27] Dibsdall L, Lambert N, Bobbin R, Frewer L (2003). Low-income consumers’ attitudes and behaviour towards access, availability and motivation to eat fruit and vegetables. Public Health Nutr.

[28] County Health Rankings and Roadmaps: Building a Culture of Health, County by County [http://countyhealthrankings.org].

[29] Huff J (2011). Lenoir County 2011 Community Health Assessment.

[30] Where Inequality Lives: The US Income Gap by County [://www.pewtrusts.org/en/research-and-analysis/blogs/stateline/2014/06/where-inequality-lives-the-us-income-gap-by-county].

[31] Sampson RJ, Raudenbush SW (1999). Systematic social observation of public spaces: a new look at disorder in urban neighborhoods 1. Am J Sociol.

[32] Brownson RC, Hoehner CM, Day K, Forsyth A, Sallis JF (2009). Measuring the built environment for physical activity: state of the science. Am J Prev Med.

[33] Viera AJ, Garrett JM (2005). Understanding interobserver agreement: the kappa statistic. Fam Med.

[34] Hillier B (2008). Space and spatiality: what the built environment needs from social theory. Building Res Inf.

[35] Swidler A. Talk of love: How culture matters. Chicago: University of Chicago Press; 2013.

